# Nucleation and Crystallization of PA6 Composites Prepared by T-RTM: Effects of Carbon and Glass Fiber Loading

**DOI:** 10.3390/polym11101680

**Published:** 2019-10-14

**Authors:** Nerea Zaldua, Jon Maiz, Amaia de la Calle, Sonia García-Arrieta, Cristina Elizetxea, Isabel Harismendy, Agnieszka Tercjak, Alejandro J. Müller

**Affiliations:** 1 POLYMAT and Polymer Science and Technology Department, Faculty of Chemistry, University of the Basque Country, UPV/EHU, Paseo Manuel de Lardizábal 3, 20018 Donostia-San Sebastián, Spain; nzaldua7@gmail.com (N.Z.); jon.maiz@polymat.eu (J.M.); 2 TECNALIA, Parque Tecnológico de San Sebastián, Mikeletegi Pasealekua 2, Donostia, E-20009 San Sebastián, Spain; amaia.delacalle@tecnalia.com (A.d.l.C.); sonia.garcia@tecnalia.com (S.G.-A.); cristina.elizetxea@tecnalia.com (C.E.); isabel.harismendy@tecnalia.com (I.H.); 3 Group ‘Materials + Technologies’ (GMT), Department of Chemical and Environmental Engineering, Faculty of Engineering, Gipuzkoa, University of the Basque Country, UPV/EHU, Plaza Europa 1, 20018 Donostia-San Sebastián, Spain; agnieszka.tercjaks@ehu.eus; 4 IKERBASQUE, Basque Foundation for Science, 48013 Bilbao, Spain

**Keywords:** thermoplastic, caprolactam, glass fibers, carbon fibers, crystallization, elastic modulus, tensile strength

## Abstract

Thermoplastic resin transfer molding (T-RTM) is attracting much attention due to the need for recyclable alternatives to thermoset materials. In this work, we have prepared polyamide-6 (PA6) and PA6/fiber composites by T-RTM of caprolactam. Glass and carbon fibers were employed in a fixed amount of 60 and 47 wt.%, respectively. Neat PA6 and PA6 matrices (of PA6-GF and PA6-CF) of approximately 200 kg/mol were obtained with conversion ratios exceeding 95%. Both carbon fibers (CF) and glass fibers (GF) were able to nucleate PA6, with efficiencies of 44% and 26%, respectively. The α crystal polymorph of PA6 was present in all samples. The lamellar spacing, lamellar thickness and crystallinity degree did not show significant variations in the samples with or without fibers as result of the slow cooling process applied during T-RTM. The overall isothermal crystallization rate decreased in the order: PA6-CF > PA6-GF > neat PA6, as a consequence of the different nucleation efficiencies. The overall crystallization kinetics data were successfully described by the Avrami equation. The lamellar stack morphology observed by atomic force microscopy (AFM) is consistent with 2D superstructural aggregates (n = 2) for all samples. Finally, the reinforcement effect of fibers was larger than one order of magnitude in the values of elastic modulus and tensile strength.

## 1. Introduction

Polyamides (PA) represent a versatile group of plastics that have been successful for 70 years in the field of engineering, textiles, transport and fibers because of their good combination of thermal and mechanical properties [1,2]. Among different polyamides, poly(ε-caprolactam), P(ε-CLa), also known as polyamide-6 (PA6) or nylon-6, has gained interest due to its high strength, high melting point, good fatigue resistance, moderate water absorption (about 8–10%), and good resistance to most common solvents and weak acids [3,4].

In addition to the traditional method of synthesis, such as polycondensation, some PAs can be synthesized by anionic ring-opening polymerization (AROP) of the corresponding cyclic lactams. The first description of CLa polymerization by AROP dates back to 1941 [2,5]. Nowadays, the ring-opening polymerization (ROP) of ε-caprolactam constitutes the most employed method to obtain PA6. Nowadays, anionic polymerization is the most industrially relevant route for the ROP of ε-caprolactam [6,7,8].

In the case of fiber composites with high fiber loadings, resin transfer molding (RTM) is commonly used, as this process satisfies the characteristics demanded by the automotive and aerospace industry [9]. This process looks for low viscosity resins, which can be injected into the mold where fibers are previously loaded. RTM allows the manufacture of large and highly complex parts [10]. Initially, this technique was dedicated to the production of thermoset resins; however nowadays, the thermoplastic resin transfer molding (T-RTM) is studied as a promising method to produce large-scale composites in automotive applications. The materials produced through T-RTM could be cheaper, safer and more recyclable, and in addition, they should be produced relatively fast due to their quick chemical reaction polymerization. So far, this quick reaction has been very difficult to control and model [11,12].

Anionic polymerization of ε-caprolactam has been employed to directly fabricate parts via thermoplastic resin transfer molding (T-RTM) [13,14,15]. This is possible since anionic polymerization occurs at a fairly fast rate (i.e., in a few minutes) and relatively low temperatures (i.e., 150 °C). This reactive processing method allows composites to be easily processed in a way similar to their thermoset counterparts, and with short mold cycle times [16]. Such polymerization has been investigated from a chemical point of view [17,18], and the influence of the polymerization conditions (type of catalyst/activator, concentration, temperature) on the process has been reported in the literature [17,19,20,21,22,23]. In addition, it has been studied that only a specific proportion of activated monomer and catalyst guarantees a complete reaction with a low amount of residual monomer [24]. Hence, there is a great interest in studying applications of T-RTM in reaction injection molding, pultrusion and vacuum infusion due to the rapid PA6 and reinforced PA6 polymerization process resulting in molded parts with excellent mechanical properties [17,25,26,27,28].

On the other hand, it has been reported in the literature that polymerization and crystallization in PA6 take place simultaneously, resulting in materials with a high degree of crystallinity in both neat PA6 and reinforced PA6 materials [29,30,31,32].

In the present work, we have used a new technology, adapted from thermoplastic resin transfer molding (T-RTM), named and patented as CAPROCAST, and developed at TECNALIA [33,34,35,36]. This process is based on the capacity of ε-caprolactam (the monomer of polyamide 6) to polymerize in situ with an anionic reactive reaction. This reaction can be carried out *in-situ* together with the polymer processing. In this work, the polymerization and filling of the mold are produced in a single stage, yielding a thermoplastic composite material with high fiber content (>60% by weight) of either fiberglass or carbon fibers.

The objective of this work is to obtain neat PA6 materials and fiber based PA6 composites by T-RTM, in order to analyze the influence of the fiber loading on: polymerization degree, molecular weight, thermal and mechanical properties. For this purpose, thermogravimetric analysis (TGA), solution viscosimetry, differential scanning calorimetry (DSC), X-ray diffraction (XRD) and tensile tests have been performed. 

## 2. Materials and Methods 

The products used for anionic ring opening polymerization of polyamide 6 (PA6) are the ε-caprolactam monomer, purchased from BASF (Rhein, Germany), sodium aluminum lactate, as initiator, and a commercial mixture of isocianates, as an activator [33,34,35,36]. The reinforcement fabrics used were glass fibers (GF) and carbon fibers (CF) fabrics.

The glass fibers were incorporated as a fabric. They consisted of a twill 2 × 2 glass fiber fabric with 600 g/m^2^ and amilosilane sizing. They were supplied by Hexcel (Hexforce 01038 1250 TF970, Dagneux, France). Four layers of glass fiber fabric reinforcement were used in each laminate panel. The resulting composites had a fiber weight content of 60%. The carbon reinforcement used was a high strength non-crimp carbon fiber fabric with 300 g/m^2^ and a binder veil incorporated. It was supplied by SGL (Sigratex C B310-45 SF-T240, Meitingen, Germany). Five layers of carbon fiber reinforcement were used in each laminate panel. The resulting composites had a fiber weight content of 47%.

**Figure 1:**
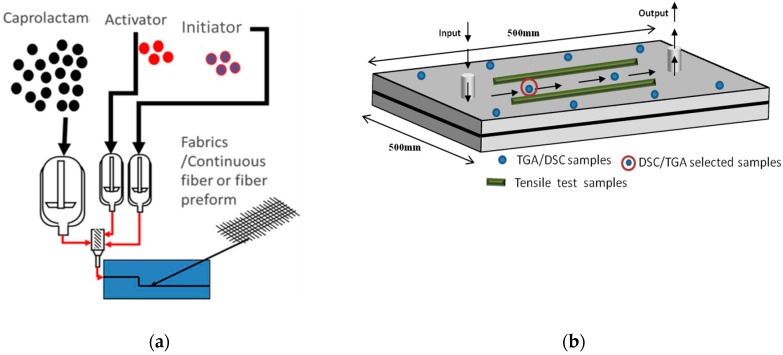
(**a**) Scheme of the T-RTM process employed. (**b**) Mold employed to obtain the PA6 and PA6/fibers composites plates. As indicated in the experimental part, 10 samples were tested by DSC and TGA (blue points in Figure 1b). As they were all identical, the DSC/TGA results reported in this paper are those obtained by samples collected from the central part, indicated in Figure 1b by a blue point surrounded by a red circle. The green rectangles mark the approximate position from which tensile bars were cut.

The CAPROCAST process developed at TECNALIA [33,34,35,36] consists of a device for polymerizing lactams directly in a mold. In a pressurized vessel connected to the mold, the reaction mixture was prepared by melting the reactants at 125 °C under stirring. The mold containing the fabrics is closed and heated to 165 °C (i.e., the polymerization temperature), and then vacuum inlets are connected to the outlet injectors to suck the air out with a vacuum pump. Once the reaction mixture is homogenous, it was injected into the mold under a pressure profile from 1 to 5 bar. The pressure was maintained during the polymerization time, and after this, the mold was allowed to cool slowly, for 8 h approximately. Figure 1a shows a scheme illustrating the T-RTM process employed to produce the PA6/fiber composite materials and Figure 1b shows a schematic description of flow, injection points, and dimensions of the mold employed for plate manufacturing. Figure 1b also shows in blue points the places from which samples were taken for TGA and DSC tests, and in green lines, the central part from which samples were cut for tensile tests.

For the subsequent characterizations, samples of PA6 reinforced with glass fiber (PA6-GF), with carbon fiber (PA6-CF) and without reinforcement were obtained.

### TGA—Monomer Conversion

Final monomer conversion and fiber content were determined by thermogravimetric analysis (TGA) in a TGA Q500 (TA Instruments, New Castle, USA) under nitrogen atmosphere. Samples of 5 mg were heated from room temperature up to 600 °C at a heating rate of 5 °C·min^−1^. It should be noticed that the samples used in TGA are the same ones used for DSC analysis (see Figure 1b). Prior to analysis, samples were dried and stored in a desiccator to prevent moisture uptake. However, the samples may have absorbed water during their transfer to the instrument, as it will be verified later on.

### Dilute Solution Viscosimetry

Since PA6 is insoluble in most common solvents, techniques like GPC cannot be used for the determination of the molecular weight. The molecular weights of the PA6 samples were determined by solution viscosimetry. The viscosity average molar mass (*M_η_*) was determined by dilute solution viscosimetry using an intrinsic Ubbelohde viscometer (SI Analytics, Mainz, Germany), with a capillary diameter of 0.53 mm. To obtain *M_η_* values the samples were dissolved in formic acid (85%) for the preparation of solutions with concentrations of 0.0015 g mL^−1^. The *M_η_*can be calculated by the Mark–Houwink Equation (1):

(1) $$\left[\eta \right]=KM_{\eta }^{\alpha }$$

where constants *K* and *α* are equal to 22.6 × 10^−3^ cm^3^·g^−1^ and 0.82, respectively, [37] and [*η*] is the intrinsic viscosity in dL·g^−1^.

### DSC—Crystallinity Characterization

Thermal behavior of the reinforced PA6 was analyzed by differential scanning calorimetry (DSC), employing a Perkin-Elmer DSC 8000 (Waltham, USA). The non-isothermal DSC scans were collected employing a first heating–cooling–heating cycle. The heating and cooling rates employed were 20 °C·min^−1^ from 25 to 250 °C under a nitrogen flow of 20 mL·min^−1^. The thermal properties of the crystalline phase after the polymerization in the mold were determined from the first heating scan, however, the second heating scan was used for an evaluation of the inherent properties of reinforced PA6 sample. 

The isothermal crystallization kinetics experiments were performed according to the procedure recommended by Lorenzo et al [38]. Samples were first cooled down from 250 °C to the selected crystallization temperatures (*T_c_*) (range of 180–196 °C) at 80 °C·min^−1^. They were kept at the selected *T_c_* values for enough time to allow the material to complete its crystallization isotherm (see Supporting Information Figure S1), i.e., approximately three times the peak time obtained in the isotherm.

Self Nucleation (SN) tests were performed according to the procedures recently reviewed by Müller et al [39,40,41,42]. This protocol is a thermal technique based on the inherent capacity of the material to form self-nuclei (either from crystal fragments or chain segments with residual crystal memory) that are the best nucleating source for any polymer. The following steps were performed with scanning rates of 20 °C·min^−^^1^: (a) erasing thermal history by heating the sample to 250 °C and keeping it at this temperature for 1 min; (b) cooling the sample from 250 °C down to 25 °C; (c) heating to a self-nucleation temperature *T_s_* at which the sample remains for 5 min; (d) cooling down from *T_s_* to 25 °C, while the cooling scan is recorded in order to monitor any changes in *T_c_* due to SN; (e) the sample is finally heated from 25 °C up to 250 °C; this final heating scan will reflect any changes in the melting of the crystals or if annealing took place.

### SAXS/WAXS—Crystalline Structure 

The samples were examined at room temperature by simultaneous small angle x-ray scattering and wide angle X-ray scattering (SAXS/WAXS) measurements at beamline BL11-NCD at the ALBA Synchrotron Radiation Facility, in Barcelona, Spain. The energy of the X-ray source was 12.4 keV (λ = 1.0 Å). In the WAXS configuration, the patterns were recorded using a Rayonix LX255-HS detector with an active area of 255 × 85 mm (pixel size: 40 µm^2^), the distance employed was 15.5 mm with a tilt angle of 27.3°. In the case of SAXS configuration, the sample detector was a Pilatus 1M (from Dectris) with an active area of 168.7 × 179.4 mm (pixel size: 172 µm^2^) and the sample-to-detector distance was 6463 mm with tilt angle of 0°, covering a scattering vector (*q*) range from 0.2–2.5 nm^−1^. The calibration was performed employing silver behenate and Cr_2_O_3_ standards. The intensity profile was output as the plot of the scattering intensity vs. scattering vector ($\;q=\;4\pi sin\theta \lambda^{-1}$).

### AFM—Morphological Observations

The morphology of the samples obtained after the T-RTM process was observed by atomic force microscopy (AFM, Bruker, Santa Barbara, USA). A Bruker 8 Multimode scanning probe microscope equipped with a Nanoscope V controller was employed for AFM. The figures were acquired in tapping mode using microfabricated silicon tips/cantilevers (cantilever spring constant, *k* = 42 N/m, and resonance frequency, *f*_0_ = 320 kHz, Bruker). Height and phase images of lamellae were collected simultaneously and were subjected to a first-order plane-fitting procedure to compensate for sample tilt. 

### Tensile Properties

Tensile tests, according to ASTM D3039 (reinforced materials) and D-638 (neat PA6) standards, were performed using an Instron machine (Norwood, USA), model 5500. Samples of (PA6-GF) and (PA6-CF) were tested at a speed of 2 mm/min. An extensometer was used to measure the elongation of the specimen in a measurement range of 50 mm. 5 specimens have been extracted from the central area (Figure 1b) of the plate with a size of 250 mm × 25 mm × 2.5 mm, and the tensile tests were done in the direction of the flow.

### TGA and DSC Results: Estimation of Errors

We first performed TGA in the Nylon/fiber composite plates, taking samples from 10 different positions within the plates (indicated in Figure 1b above). The results were highly reproducible with errors in weight measurements within 1% and in temperatures of less than 0.4 °C variations.

We did similar measurements with DSC. Once again highly reproducible results were obtained regardless of the location of the sample. The changes in *T_c_* and *T_m_* values were below 0.3 °C, so they are well within the typical DSC temperature errors (usually about 0.5 °C when calibration and baseline drifts are taken into account). In the case of latent enthalpies of crystallization and melting, the errors were less than 10%, once again a typical value for DSC measurements when the integration error is taken into account together with baseline drift and calibration.

Having checked that sample reproducibility was excellent irrespective of sample positioning in the plates, and within the errors of the TGA and DSC experiments, we decided to report in this paper measurements performed to samples taken from the central part of the plates (as indicated in Figure 1b). The same sample (per material employed) was used for TGA and DSC measurements.

## 3. Results and Discussion

### Degree of Conversion and Molecular Weight

A full degree of conversion (*X_p_)* after polymerization is a fundamental parameter in order to produce materials with the best possible properties and minimum toxicity. As conversion increases in the mold, long-chain PA6 molecules are produced and the viscosity of the melt increases hindering the diffusion of the monomers to the active growing chains. Therefore, it is expected that a small amount of residual monomer remains at the end of polymerization. The residual monomer has a plasticizing effect on the mechanical properties of the PA6 or the PA6 matrix (in the case of composites). Therefore, it is of utmost importance to determine the degree of conversion, and in our case, this was determined from the remaining amount of ε-caprolactam (CL) in neat PA6, PA6-GF and PA6-CF detected by TGA experiments. 

**Figure 2:**
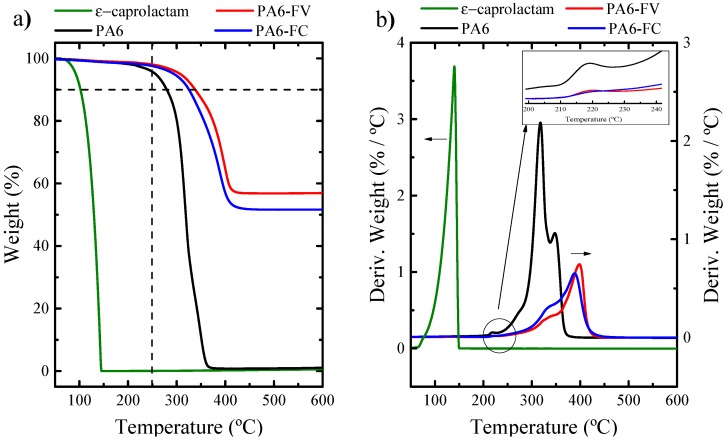
TGA results, expressed as weight as a function of temperature (**a**) and its derivative (**b**) for neat PA6, the indicated composites and for the ε-caprolactam monomer at 5 °C·min^−1^.

Figure 2 shows the TGA results obtained at a rate of 5 °C·min^-1^. Under these conditions, the samples degrade in two distinctive ranges. The first, small but significant, weight loss is observed in the range of 100–250 °C and this is due to evaporation of the ε-caprolactam and to a minor contribution of water evaporation [22]. This result was corroborated by measuring the neat monomer employed (ε-caprolactam), also represented in Figure 2a. In Figure 2b, these small mass losses can be better appreciated by changes in the derivative of the weight loss curve represented also in a close-up inset. The second and most important weight loss range starts at approximately 250 °C, where PA6 begins to degrade. 

Comparing the neat and the composite polymeric samples in Figure 2, the improvement in thermal stability of PA6 due to fiber incorporation is evident. Table 1 provides quantification of several important parameters derived from the data reported in Figure 2 [43]. It is worth comparing the temperature at which the samples lose 10% of their weight in Table 1. In the case of neat PA6, this occurs at a temperature of 282 °C, while for reinforced materials it happens at significantly higher temperatures, i.e., 312 and 358 °C, for PA6 with carbon and glass fibers, respectively.

Comparing the neat and the composite polymeric samples in Figure 2, the improvement in thermal stability of PA6 due to fiber incorporation is evident. Table 1 provides quantification of several important parameters derived from the data reported in Figure 2 [43]. It is worth comparing the temperature at which the samples lose 10% of their weight in Table 1. In the case of neat PA6, this occurs at a temperature of 282 °C, while for reinforced materials it happens at significantly higher temperatures, i.e., 312 and 358 °C, for PA6 with carbon and glass fibers, respectively.

In order to define the residual monomer ratio, the weight loss derivative was used to identify the inflection point between monomer evaporation and polymer degradation, in the range of 250 °C (observe the enlarged part of Figure 2b). According to this, the CLa conversion of neat PA6 and reinforced PA6 was determined using the proportion between the remaining weight of ɛ-caprolactam (*m_CLa_)* and the total mass of sample (*m*_0_) according to Equation (2).

(2) $$X_{p}=1-\frac{m_{CLa}}{m_{0}}$$

All the samples possessed a CLa conversion higher than 95%, considering the uncertainty that this measurement may present, around 2%, as shown in Table 1. These values are in agreement with literature data obtained by anionic polymerization [24,44,45] and correspond to a practically complete transformation as the monomer-polymer equilibrium does not permit a 100 wt.% conversion of ε-caprolactam [46].

So, our conditions of in situ polymerization lead to a high degree of conversion for both neat and reinforced PA6 samples. This indicates that any differences that may be observed in the properties of the matrix do not correspond to a low conversion in the samples. 

The degradation trends in nitrogen environment for PA6, PA6-GF and PA6-CF composites are similar, but the residues left behind after decomposition were different. While the residue left is about 0% in the case of PA6, it was found to be 57% for PA6-GF and 51% for PA6-CF composites under N_2_. This residue corresponds to the fiber content, and it is in agreement with the nominal fiber content employed and reported in Table 1 corresponding to the fiber content of the sample in weight %.

Finally, the mechanical properties of the PA6 composites may also be affected by the molecular weight of the matrix. To study the effects of molecular weight, the viscosity-average molecular weights (*M_η_*) of neat PA6 and the PA6 matrices in the reinforced composites were measured, as explained in the experimental part. The results are listed in Table 1, and as can be seen, the values obtained are relatively high, around 200 kg/mol, which is suitable for industrial applications. The molecular weight of neat PA6 and PA6 matrix is similar in both composite samples. We can, therefore, conclude that the presence of the fibers does not affect the molecular weight of the matrix during the *in-situ* polymerization process in the mold. 

### DSC—Thermal Properties and Crystallinity Degree

It should be noted at this point that the synthesis of PA6 is carried out at 165 °C, which is also a temperature at which PA6 crystallizes. As previously mentioned, the crystallization process occurs simultaneously during the polymerization of ε-caprolactam, so this process can affect the crystalline phase of the PA6. DSC studies were performed on all the samples to investigate the thermal properties related with the crystalline phases of the materials.

**Figure 3:**
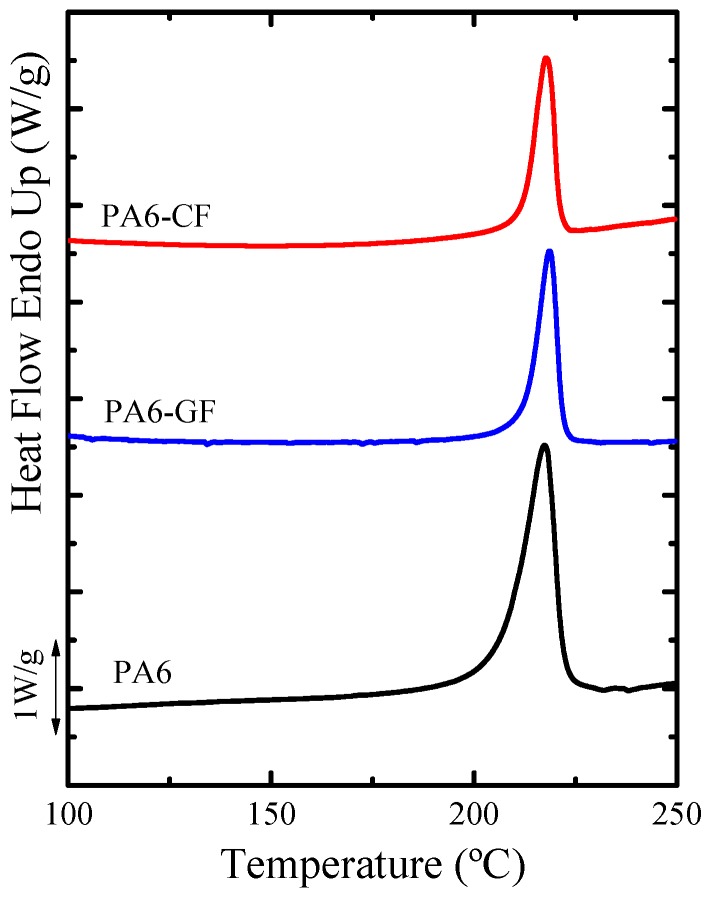
First DSC heating scans for neat PA6 and the composites with glass fiber (GF) and carbon fiber (CF). Scans presented in the image are not standardized by the weight fraction of PA6.

Figure 3 shows the first DSC heating scans of PA6 and its composites, where the results obtained from this first scan exhibit the properties of the untreated material after the synthesis process. Only one melting peak at high temperatures is observed for all the samples studied, where the lack of evidence of a cold crystallization peak during the first heating scans indicates that in all cases the PA6 matrix has been able to crystallize during the polymerization process and its subsequent slow cooling in the mold. It is clear from Figure 3 that there were no significant changes in the melting temperatures of the polyamides with or without fibers, which are around 218–219 °C for neat PA6, PA6-GF and PA6-CF. Furthermore, Table 2 reports the enthalpies of melting (Δ*H_m_*) and crystallinity degree (*X_c_*) of neat PA6 and PA6 with GF and CF, respectively. The enthalpy values obtained from the first heating scans for both PA6 composites are slightly higher than that obtained for neat PA6. 

The crystallinity of PA6 matrix can be calculated using the following Equation (3):

(3) $$X_{c}\left(\%\right)=\frac{\Delta H_{m,cor}}{\Delta H_{100\%}}$$

where Δ*H* for 100% crystalline PA6 is 188 J/g and Δ*H_m,cor_*is the measured enthalpy corrected by the weight fraction of PA6 or ΔHm(1−α) where α is the fiber fraction calculated by TGA (Table 1). 

The DSC determined crystallinity for both composites is slightly higher than for neat PA6, as it can be observed in Table 2. This difference is not really significant (almost within the experimental error of the measurements, which is in the order of 10%) since the cooling conditions inside the mold have been slow and therefore the matrix has had the capacity to slowly crystallize to its maximum achievable degree under the applied conditions.

**Figure 4:**
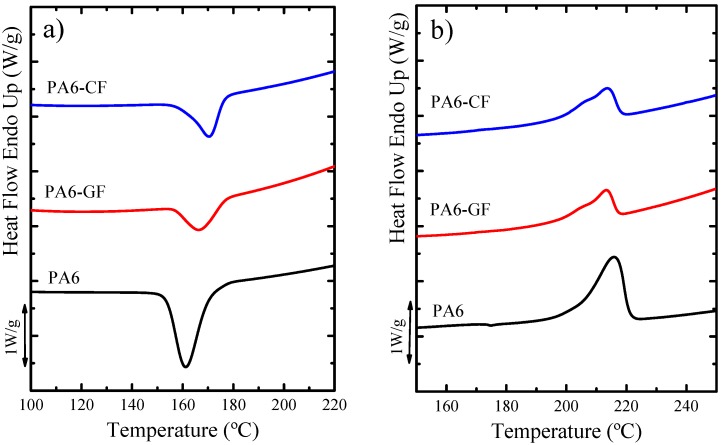
DSC (**a**) cooling and (**b**) heating curves of polyamide 6 and polyamide 6 composites at 20 °C·min^−1^ rate. Scans presented in the image are not standardized by the weight fraction of PA6.

After the cooling data was collected, and once the crystalline thermal history of the material was erased, the subsequent DSC heating scans were recorded, and previously formed crystals at cooling rates of 20 °C min^−1^ were melted in order to analyze the effect of the reinforcement on the PA6 matrix, without any polymerization effects in the samples. Figure 4 and Table 2 present the DSC scans and the data obtained from the non-isothermal DSC runs, respectively.

In Figure 4a, the non-isothermal crystallization of the samples at 20 °C·min^−1^ is shown. The peak *T_c_* values displayed in Table 2 confirm that the composites have higher crystallization temperature than neat PA6. This indicates that under non-isothermal cooling conditions, both GF and CF can nucleate the PA6 matrix, where in the case of the CF the nucleating capacity is higher since its *T_c_* value is 170.5 °C, whereas for the GF composite is 166.6 °C. These values should be compared with that of neat PA6, whose peak crystallization temperature is 161.3 °C. The peak crystallization temperature upon cooling from the melt corresponds to the temperature at which the crystallization rate goes through a maximum and spherulites (or axialites) impinge on one another, hence it can be correlated with the maximum nucleation density of the material. 

In the DSC heating scans, only the melting behavior endotherms are observed (see Figure 4b) for all the samples. The difference in the melting peak temperatures between the samples is not very large. However, the *T_m_* values observed during the first heating scans are somewhat higher (3–4 °C) than those observed for the corresponding sample during the second heating scan, as can be seen in Table 2. The reason for this difference is due to their different thermal histories. The samples obtained just after polymerization crystallized during slow cooling and are expected to have crystallized at higher *T_c_* values, hence they formed thicker lamellar crystals on average, which melt at higher values. Also, the slow cooling of the samples leads to higher crystallinity degrees as expected (see Table 2).

### SAXS/WAXS—Crystalline Structure

The crystallization of the PA6 component during the T-RTM process was also confirmed by simultaneous SAXS/WAXS analyses of PA6 without and with glass fiber (PA6-GF) and carbon fiber (PA6-CF).

**Figure 5:**
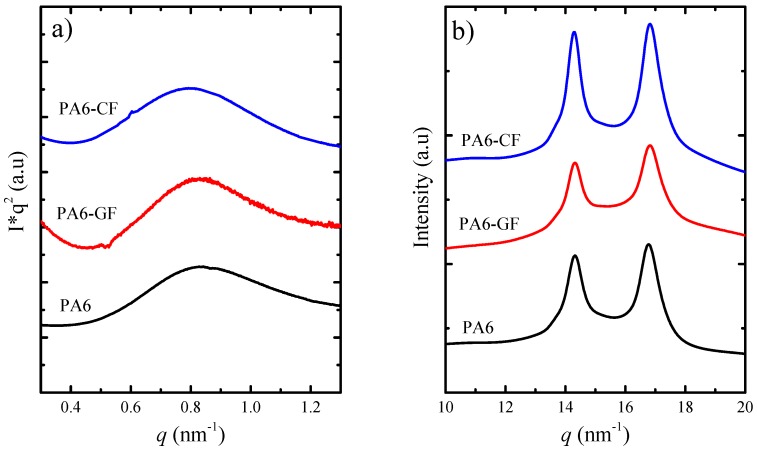
(**a**) SAXS (Small Angle X-ray Scattering) patterns taken at all the PA6 samples as obtained. (**b**) WAXS (Wide Angle X-ray Scattering) diffractograms taken at the same conditions.

Figure 5a shows the SAXS measurements performed on the reinforced PA6 and PA6 samples as obtained from the mold. In all the samples a clear and relatively intense maximum is observed that is related to the lamellar stacks (whose presence was seen in real space by AFM, see below), and from those values of *q_max_*, the long periods values (d*) were calculated by Equation (4) from the corrected Lorentz plots ($I\cdot q^{2}$ versus q, Figure 5a),

(4) $$d^{*}=\;\left(2\pi \right)/q_{max}$$

The lamellar thickness can be calculated from the long period values obtained by SAXS and the degrees of crystallinities, using the approximation: *l* = X_c_ d*, where *X_c_* is the crystalline fraction [47]. We have calculated the *X_c_* from the enthalpy of melting, obtained during the first DSC heating scan (see Table 2).

The lamellar thicknesses obtained by SAXS/DSC are: 3.36, 3.54, 3.88 nm for PA6, PA6-GF and PA6-CF, respectively. These values are very similar to one another, considering the error involved in the measurements (typically 10%), so they can all be approximated to 3.5 nm. Therefore, the presence of the fibers did not affect the lamellar thickness of the crystals in the conditions of slow cooling used during the polymerization/crystallization simultaneous process in the mold. In addition, the values of *l* are proportional to the melting temperature of the samples, through the Gibbs–Thomson equation [47]. Our first DSC heating scans for the samples showed that the *T_m_* values are similar in all samples. Therefore, both DSC and SAXS results are consistent.

Figure 5b shows the WAXS patterns taken after the T-RTM process. The main WAXS reflections are observed for all samples and no changes in crystalline structure is detected. 

The main reflection peaks of PA6 are located at *q*-values of 15.2 and 16.8 nm^−1^, and correspond to the (200) and (002)/(202) planes, respectively—all of them being characteristic peaks of the alpha PA crystalline form. It is worth noting that the characteristic shoulder usually observed in the PA6 at 15.7 nm^−1^ does not appear in both neat PA6 and in the composites and corresponds to the (001) plane. All the reflections are consistent with the reported monoclinic unit cell of PA6 with unit cell parameters *a* = 9.56, *b* = 8.01, and *c* = 17.24 Å [48,49].

It should be noted that the lack of a characteristic peak at 15.7 nm^−1^ is a clear sign of the absence of the γ crystal structure. Therefore, the only crystalline structure present in all the samples is the α phase, which is considered the most stable crystalline structure for PA6 and the one leading to the best mechanical properties.

### Self-Nucleation (SN)

To corroborate the efficiency of the fibers as nucleating agents, it is necessary to compare their effectiveness with PA6 self-nuclei. In theory, the best nucleating agents for any polymer are their own crystal fragments or chain segments with residual crystal memory [39,40,50]. With this objective, the self-nucleation thermal protocol was applied for the production of self-nuclei within the polymer melt, thereby increasing dramatically the nucleation density of PA6. 

**Figure 6:**
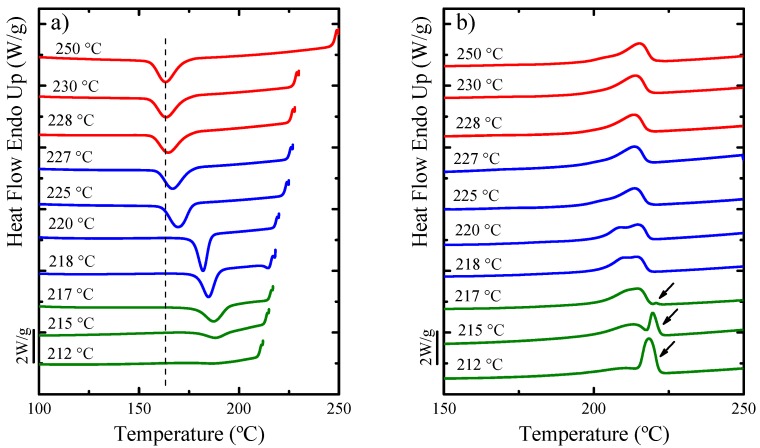
(**a**) DSC cooling scans for neat PA6 after 5 min at the indicated *T_s_* values and (**b**) subsequent heating scans after the cooling runs shown in (**a**). The colors indicate the different self-nucleation *Domains*: red for *Domain I*, blue for *Domain II* and green for *Domain III*. The dashed vertical line in (**a**) has been drawn to indicate the standard peak crystallization value for PA6.

Figure 6 shows the results obtained during an SN experiment for neat PA6. The cooling scans after the isothermal steps at *T_s_* are presented in Figure 6a, and the subsequent heating scans are shown in Figure 6b, where the presence of the three SN *Domains* are evident [42,50]. 

The polymer is in *Domain I* when complete melting occurs, and the crystalline history of the material is erased. For PA6 *Domain I* is found at *T_s_* larger or equal to 228 °C since no change is detected in *T_c_* when compared to the standard *T_c_*, namely, the crystallization and melting temperatures are identical in *Domain I*. These scans are represented with red lines in Figure 6.

A cross-over to *Domain II* or self-nucleation *Domain* occurs when the *T_s_* range employed is low enough to produce self-nuclei, but high enough to avoid annealing. Therefore, *Domain II* can be identified when the peak *T_c_* of the sample increases as compared to the standard value. For PA6, the sample is in *Domain II* in the following range, *T_s_* = 227–218 °C. The minimum *T_s_* value within *Domain II* is defined as the “ideal self-nucleation temperature” (*T_s_*, ideal), a temperature that must be accurately determined. This ideal value is the *T_s_* temperature that causes maximum self-nucleation without annealing, which is 218 °C, see Figure 6a. The subsequent melting curve for the *T_s_* = 218 °C in Figure 6b does not reveal any sign of annealing, confirming that this is the *T_s,ideal_*. In *Domain II*, the nucleation density is enhanced exponentially as *T_s_* decreases, which makes the crystallization of PA6 possible at higher temperatures. The peak crystallization temperature is proportional to the nucleation density under non-isothermal conditions. The *Domain II* scans are represented with blue lines in Figure 6.

Finally, when *T_s_* is too low to completely melt the polymer and only partial melting occurs (during the 5 min holding time at *T_s_*), the unmolten crystals anneal giving rise to *Domain III* or the self-nucleation and annealing *Domain*. Figure 6b shows that when *T_s_* < 218 °C, the melting endotherms show high-temperature second peaks because of the melting of annealed crystals, which reveal that the sample is in *Domain III*. These scans are represented with green lines in Figure 6. The melting peaks of annealed crystals are signaled by arrows in Figure 6b [42,50]. 

The *T_c_*corresponding to the ideal *T_s_* (i.e., 218 °C) can be used as the maximum crystallization temperature (*T_c,max_*) achieved in *Domain II*, in order to determine the nucleation efficiency of the nanofillers. The *T_c,max_* used in this study for the neat PA6 sample was 184.6 °C.

The nucleation efficiency (NE) of glass fiber or carbon fiber on the PA6 matrix can be determined with Equation (5), proposed by Fillon et al [51].

(5) $$NE=\frac{T_{c,PA6+fiber}-T_{c,PA6}}{T_{c,max}-T_{c,PA6}}$$

where *T_c,PA6+fiber_* is the peak crystallization temperature of the polymer with the corresponding fiber (values given in Table 2), *T_c,PA6_* is the peak crystallization temperature of neat PA6 (value Table 2) and *T_c,max_* is the maximum crystallization temperature of the ideally self-nucleated neat PA6, as mentioned before.

The nucleating efficiency is higher when carbon fibers are added than when glass fibers are added. In the case of carbon fibers, the nucleation efficiency is 43.5% and for glass fibers only 26.1%. The increase in nucleation efficiency is less than expected due to the mesh shape distribution of the fibers. The material does not act as an efficient nucleation agent in the whole matrix, only in the parts that have contact with the fibers. Although after nucleation at the surface of the fibers, the crystallization spreads by secondary nucleation. Even so, the process is not as efficient, as if the fibers were well dispersed in the polymer matrix.

### Overall Isothermal Crystallization Studied by DSC

Figure S1 (Supporting Information) shows the isothermal crystallization exotherms of neat PA6 and for the glass and carbon fiber composites. It is seen that for all the samples the crystallization exothermic peak becomes flatter, and the time to reach the maximum degree of crystallization increases, as the crystallization temperature increases, as expected [52]. 

The half-time of crystallization, *τ_50%_*_,_ is an important parameter for the analysis of crystallization kinetics because it measures the time needed to reach a relative conversion of 50%. Therefore, an experimental measure of the overall crystallization rate that includes both nucleation and growth, is the inverse of the half-crystallization time, determined by DSC isothermal crystallization experiments [52].

**Figure 7:**
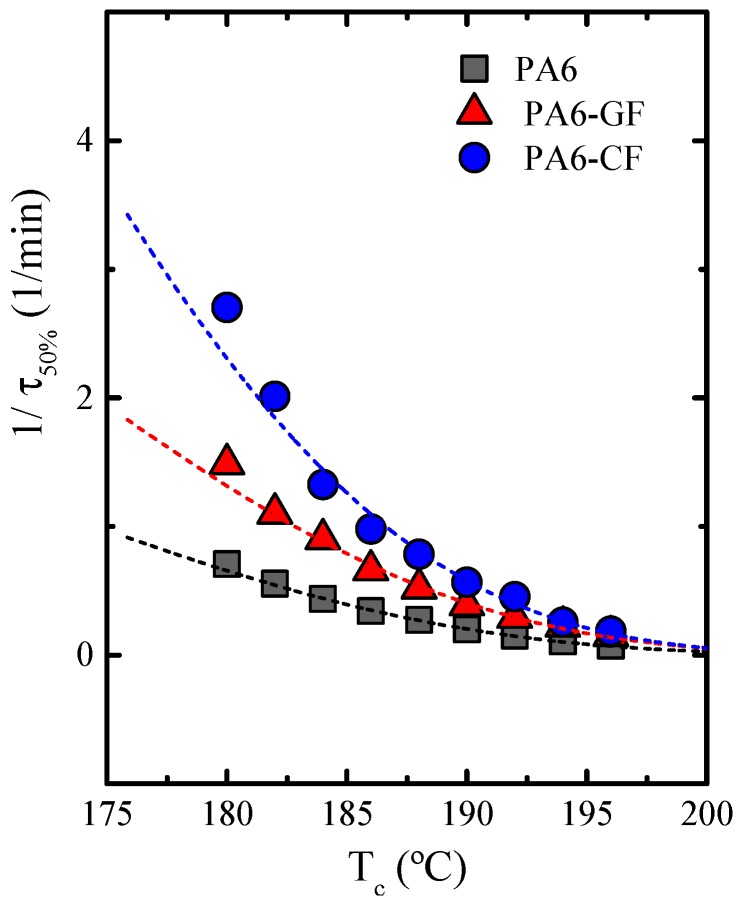
Inverse of the half-crystallization time as a function of crystallization temperature, for the indicated samples, neat PA6 and the PA6-GF and PA6-CF fiber composites. The solid lines correspond to fits of the LH model.

Figure 7 shows the inverse of the half-crystallization time (*1/τ_50%_*), as a function of the isothermal crystallization temperature (*T_c_*) for neat PA6 and its fiber composites.

Having neat PA6 as reference material, the crystallization rate increases in the composites materials, due to the nucleation effect of the fibers. Another way to examine the results presented in Figure 7 is by taking the *1/τ_50%_* values at a constant *T_c_* (i.e., 180 °C), where the obtained values are 0.71, 1.45 and 2.70 min^−1^. These results corroborate the higher crystallization rate induced by fiber addition. Carbon fibers induce a faster overall crystallization rate as a consequence of their higher nucleation efficiency. The results presented in Figure 7 are consistent with the non-isothermal crystallization results presented above.) 

**Figure 8:**
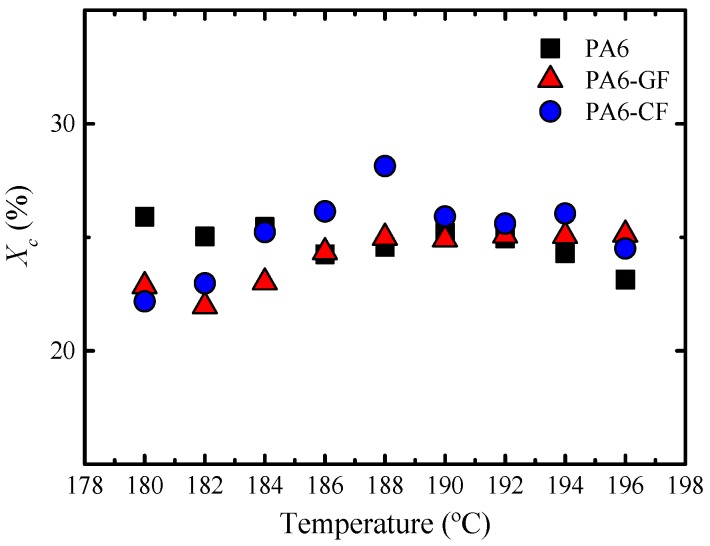
Relative crystallinity degree values (*X_c_*) as a function of *T_c_* after isothermal crystallization of PA6 and the reinforced samples.

Figure 8 plots the crystallinity values obtained during the isothermal crystallization. They were determined from the latent heat of melting of the samples, measured during non-isothermal DSC heating runs, performed from *T_c_* (after the sample had completed its isothermal crystallization process) up to the complete melting of the samples. These values can have errors of around 15% when the integration error is considered together with baseline drifts and equipment calibration.

As seen in Figure 8, the values of *X_c_* in all cases are around 25%. If we compare this value with those obtained after non-isothermal crystallization, reported in Table 2, it can be appreciated that similar values were obtained after cooling the samples with a cooling rate of 20 °C·min^−1^. Therefore, in both these conditions, the material crystallizes up to the same amount. However, these values are much smaller than those obtained by any of the samples, just after they have been prepared (by bulk polymerization and crystallization during slow cooling). As mentioned previously, this is due to the slow cooling that occurs in the mold during the T-RTM process.

### Fitting Isothermal Data to the Avrami Model

The Avrami equation [53,54,55] can be used to analyze the isothermal crystallization data of PA6 and its fiber composites. Lorenzo et al. express the Avrami equation taking into account the incubation time, i.e., Equation (6) [38,55].

(6) $$1-V_{c}\left(t-t_{0}\right)=e^{-K{\left(t-t_{o}\right)}^{n}}$$

where *t* is the experimental time and *t*_0_ is the induction or incubation time, so (*t* − *t*_0_) denotes the real-time of crystallization. *V_c_* is the relative volumetric transformed fraction, *n* is the Avrami exponent whose value depends on the mechanism of nucleation (sporadic or instantaneous) and on the dimensionality of the crystals formed (2D or 3D crystalline aggregates being the most common in polymers), and *K* is the Avrami rate constant containing both nucleation and growth contributions. The data is fitted to the Avrami equation by employing the free Origin plugin developed by Lorenzo et al. [38].

**Figure 9:**
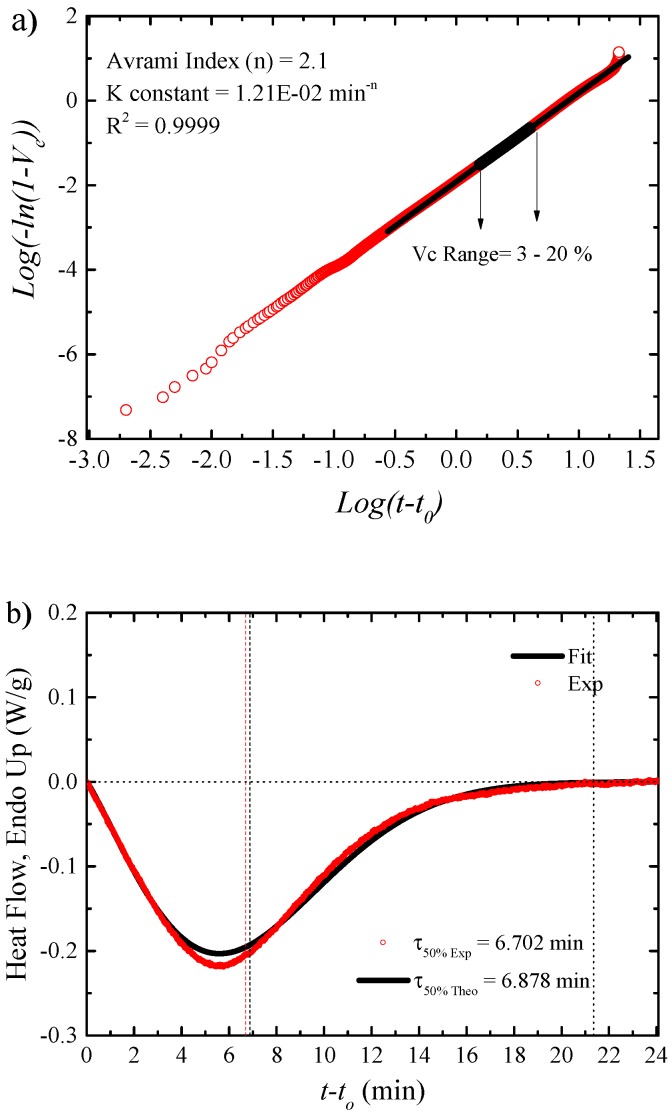
Isothermal crystallization studied by DSC. (**a**,**b**) Avrami plot and raw heat flow data obtained during crystallization of PA6 at 192 °C, compared to the data predicted by the Avrami model.

Figure 9 shows one example of the results obtained for the isothermal crystallization of neat PA6 at an isothermal crystallization of 192 °C and the fit to the Avrami model. 

Figure 9a shows the Avrami plot or double logarithmic representation that linearizes Equation (6). The beginning of the range between 3–20% is well within the primary crystallization, where free superstructural growth occurs. The Avrami equation was deduced for free growth and fits better the results in the primary crystallization regime for most polymers. The inset of the Avrami plot includes the values of the Avrami index (*n*), the constant *K* and the correlation coefficient *R*^2^. As the fit is made to a double logarithmic equation, good fits are only obtained when *R*^2^ values in excess of 0.9990 are obtained. Taking into account these values and applying them in equation 5 and differentiating the equation, it is possible to model the experimental heat flow by the Avrami Equation (6), as shown in Figure 9b.

As already mentioned, the Avrami equation normally fits well during the primary crystallization range. However, in the case of all the PA6 samples used here, the Avrami equation fits almost perfectly the DSC experimental data up to 40% conversion (see an example in Figure 9b), then the fit deviates slightly when the exotherm peaks and then a good fit of the secondary crystallization data is exceptionally obtained (for conversions exceeding 50%, after superstructural impingement). Figure 9b also shows both the experimental crystallization time and those obtained by the Avrami fit; in this example, they only differ by 3%.

**Figure 10:**
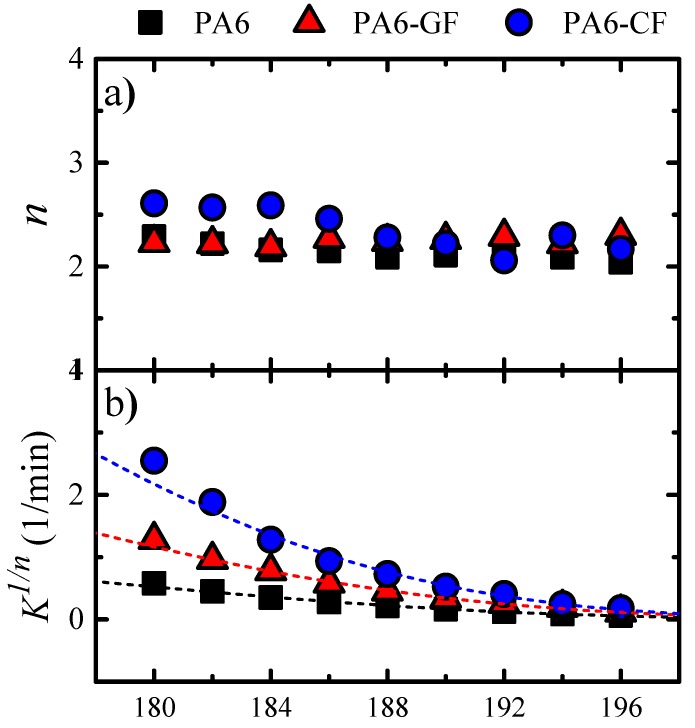
(**a**) Avrami index for the indicated samples as a function of *T_c_*. (**b**) Normalized crystallization constant of the Avrami model as a function of *T_c_*. Solid lines in (**b**) correspond to fittings to the Lauritzen–Hoffman model.

A summary of all crystallization kinetics data and the parameters extracted from the Avrami fits are plotted in Figure 10 (the data is tabulated in the Supplementary Information, see Table S1). 

Figure 10a shows *n* values as a function of *T_c_* for all samples [38,55]. The values are almost constant regardless of the isothermal crystallization temperature. The *n* values of neat PA6 vary from 2.29 to 2.04 in the investigated *T_c_* range, and those of the PA6-GF and PA6-CF composites range between 2.30–2.19 and 2.6–2.17, which is an expected result for PA6. The nucleation process is heterogeneous and instantaneous under the experimental conditions applied, which is commonly observed in PA homopolymers, and in all the samples in our case [10]. The Avrami index close to 2 indicates that axialites are formed (i.e., 2D lamellar aggregates), as a consequence of the very high nucleation density present in the samples, which prevent the axialites development into 3D spherulites superstructural aggregates. So, all the samples crystallized by forming instantaneously nucleated axialites (corresponding to *n* = 2). Attempts were made to try to visualize the crystalline morphology of microtomed samples by Polarized Light Optical Microscopy (PLOM), but we were only able to observe birefringent dots at the highest possible magnification employed (500×), attesting for the high nucleation densities that typically lead to axialites being predominant.

**Figure 11:**
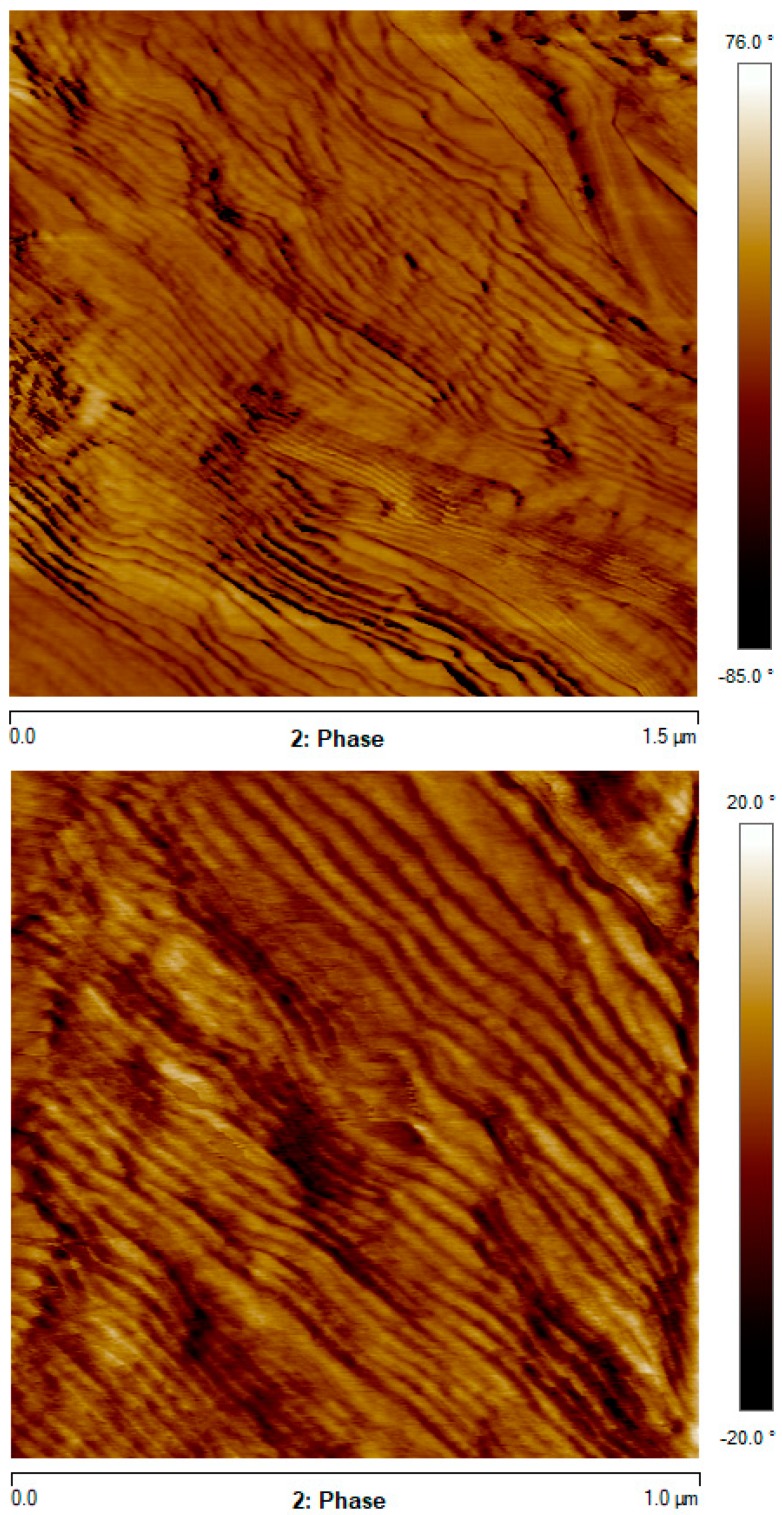
Two representative AFM phase images of neat PA6 taken from the surface of ultra-microtomed samples.

The presence of lamellar stacks and absence of 3D spherulites was also observed by AFM. The samples were ultra-microtomed at room temperature from as polymerized/crystallized plates. Figure 11 shows the typical lamellar stacks morphology observed by AFM. As the lamellae have a small tilt and are not perfectly edge-on, measurements of average lamellar thickness may contain a high error. Since they were already measured by SAXS, we prefer to use the SAXS value, as it is also more representative of averaging a larger lamellar volume. Similar images were taken from the matrix part of the nanocomposites, but the presence of fibers makes difficult the ultra-microtoming of the samples and the images obtained do not have good quality.

Figure 10b shows how the “normalized” Avrami rate constant varies with *T_c_* values. As *K* values have units of min^−n^ (i.e., the units are a function of the *n* values), it can be normalized by elevating *K* to the power 1/n. In this way, *K*^1/*n*^ has units of min^−1^ regardless of *T_c_* values and hence values for different samples can be compared.

The way *K*^1/*n*^ values vary with *T_c_* in Figure 10b is qualitatively very similar to the experimental data reported in Figure 7 and expressed as *1/τ_50%_* for neat PA6 and also for the fiber composites. Since *K* is also proportional to the overall crystallization kinetics (including nucleation and growth terms), the identical trends shown in Figure 10b and Figure 7 also corroborate the good fit of the Avrami equation to the experimental data. The values of *R*^2^ in Table S1 are always at least 0.999.

### Tensile Properties

The mechanical properties of neat PA6 and the PA6/fiber composites may be affected by the molecular weight, crystallinity degree and by the type of crystalline phase formed by PA6. In addition, the nucleating effects of the fibers could also have an effect. It is worth mentioning that the nucleation of the fibers and crystallinity degrees could also be affected by the formulation of the caprolactam (activator and catalyst) and by the sizing on the surface of the fibers (as strong interfacial bonding is desired). Other possible factors that could induce poor mechanical properties are incomplete wetting of the fiber by the polymer, presence of holes, etc. Therefore, measuring tensile properties and comparing them with similar results obtained in comparable composites is desirable to check for the quality of the T-RTM process employed to prepare the samples.

In the present work, we have observed that the PA6 matrix in all cases has a similar molecular weight (Table 1), a similar degree of crystallinity and, identical α-phase crystals. Therefore, these three properties should not cause differences in mechanical properties. On the other hand, when composites contain large amounts of reinforcing intermeshing fibers, it is the filler that dominates the mechanical properties of the composites, if good specimens have been prepared (with complete filling, high conversion, no holes and good matrix fiber interactions).

The results of these mechanical tests are shown in Table 3. They are highly satisfactory and compare well with values reported in the literature for similar composites [56].

Table 3 shows the reinforcing effect of glass and carbon fiber on the PA6 at room temperature. The addition of glass or carbon fibers improves the elastic modulus of PA6 significantly. A similar effect is produced on tensile strength, whose value greatly increases. The increases obtained in mechanical properties represent more than one order of magnitude for both modulus and tensile strength, but the reinforcing effect of CF is higher than GF, even when the amount of CF added is lower. This is expected, as the properties are dominated by the intrinsic mechanical properties of the reinforcing fibers, and it is well known that carbon fibers exhibit higher mechanical strength than glass fibers [56]. However, carbon fibers are more expensive. As lower amounts of carbon fibers are needed to obtain better properties, a balance between enhanced mechanical properties and cost can be achieved.

## 4. Conclusions

Using optimized conditions of T-RTM, neat PA6 and PA6/fiber composites were prepared, containing 60 and 47 wt.% of GF and CF, respectively. Neat PA6 and PA6 composites matrices of approximately 200 kg/mol were obtained with conversion ratios exceeding 95%. 

Both CF and GF can nucleate PA6, with efficiencies of 44% and 26% respectively in comparison to PA6 self-nuclei. The PA6 α crystal polymorph is the only one in all samples. As the cooling rate during the polymerization/crystallization/molding T-RTM process is slow, the long periods, lamellar thickness and crystallinity degree values did not show significant variations in the samples with or without fibers. 

The overall isothermal crystallization rate depends on the nucleating ability of the reinforcing fillers and decreased in the order: PA6-CF > PA6-GF > neat PA6, as a consequence of the different nucleation efficiencies of CF and GF. The overall crystallization kinetics data are successfully described by the Avrami equation with a second-order kinetics (n = 2). The absence of spherulites and presence of axialites was corroborated by AFM. The lamellar stack morphology observed by AFM is consistent with 2D superstructural aggregates (n = 2) for all samples. 

Finally, the reinforcement effect of fibers was larger than one order of magnitude in the values of elastic modulus and tensile strength. The best mechanical properties were obtained with CF (in comparison with GF) as they transfer their higher modulus and tensile strength (in comparison to GF) to the composite. 

## Figures and Tables

**Table 1:** Properties of neat PA6 and the fiber composites prepared in this work obtained by TGA and dilute solution viscometry.

	Grammage	Nominal Fiber Content wt.%	*M_η_* (kg/mol)	*X_p_* (%)	*T*_10%_ (°C)	TGA Residue (α) (%)
**PA6**	-	-	205	97	278	1
**PA6-GF**	600	60	195	98	338	57
**PA6-CF**	300	47	196	97	325	51

**Table 2:** DSC-derived data for neat PA6 and its composites with glass and carbon fiber.

	1st Heating						Cooling and 2nd Heating				
	*T_m_* (°C)	Δ*H_m_* (J/g)	ΔH_m,cor_ * (J/g)	*X_c_* (%)	*T_c_* (°C)	Δ*H_m_* (J/g)	Δ*H_c,cor._* (J/g)	*T_m_* (°C)	Δ*H_m_* (J/g)	Δ*H_m,cor._* (J/g)	*X_c_* (%)
**PA6**	217.8	81.0		43	161.3	46.2		214.2	45.6		23
**PA6-GF**	217.9	37.8	87.9	47	166.6	20.4	47.4	213.2	21.4	49.7	26
**PA6-CF**	218.6	46.5	94.9	51	170.5	24.6	50.2	213.5	25.8	52.6	28

* Δ*H_m,cor_* is the measured enthalpy corrected by the weight fraction of PA6.

**Table 3:** Results of tensile tests for neat PA6 and reinforced PA6 with glass (GF) and carbon fibers (CF). Average values and standard deviations (SD) are also reported.

	% Fiber (in wt.%)	Elastic Modulus (GPa)	Tensile Strength (MPa)
**PA6**	-	0.8	48
**PA6-GF**	60	17.3	337
**SD**	-	0.18	12
**PA6-CF**	47	44	620
**SD**	-	4.9	92

## Supplementary Materials

The following are available online at https://www.mdpi.com/2073-4360/11/10/1680/s1, Figure S1: Isothermal crystallization exotherms of neat PA6 and for the glass and carbon fiber composites, Table S1: Data obtained using Avrami equation.
