# Supplementary material for: Nucleation and Crystallization of PA6 Composites Prepared by T-RTM: Effects of Carbon and Glass Fiber Loading
Source: Polymers (Basel). 2019 Oct 14;11(10):1680. doi: 10.3390/polym11101680 (PMC6835280; doi:10.3390/polym11101680)
Supplement: Supplementary file 1 [file polymers-11-01680-s001.pdf]

Supporting Information

Nucleation and Crystallization of PA6 Composites Prepared by T-RTM: Effects of Carbon and Glass Fiber Loading

Nerea Zaldua [1], Jon Maiz [1], Amaia de la Calle [2], Sonia García-Arrieta [2], Cristina Elizetxea [2], Isabel Harismendy [2], Agnieszka Tercjak [3] and Alejandro J. Müller [1,4,*]

1 POLYMAT and Polymer Science and Technology Department, Faculty of Chemistry, University of the Basque Country, UPV/EHU, Paseo Manuel de Lardizábal 3, 20018 Donostia-San Sebastián, Spain; nzaldua7@gmail.com (N.Z.); jon.maiz@polymat.eu (J.M.)
2 TECNALIA, Parque Tecnológico de San Sebastián, Mikeletegi Pasealekua 2, Donostia, San Sebastián, E-20009 Spain; amaia.delacalle@tecnalia.com (A.d.l.C.); sonia.garcia@tecnalia.com (S.G-A.); cristina.elizetxea@tecnalia.com (C.E.); isabel.harismendy@tecnalia.com (I.H.)
3 Group 'Materials + Technologies' (GMT), Department of Chemical and Environmental Engineering, Faculty of Engineering, Gipuzkoa, University of the Basque Country, UPV/EHU, Plaza Europa 1, Donostia-San Sebastián 20018, Spain; agnieszka.tercjaks@ehu.eus (A.T.)
4 IKERBASQUE, Basque Foundation for Science, Bilbao, Spain
* Correspondence: alejandrojesus.muller@ehu.es (A.J.M.)

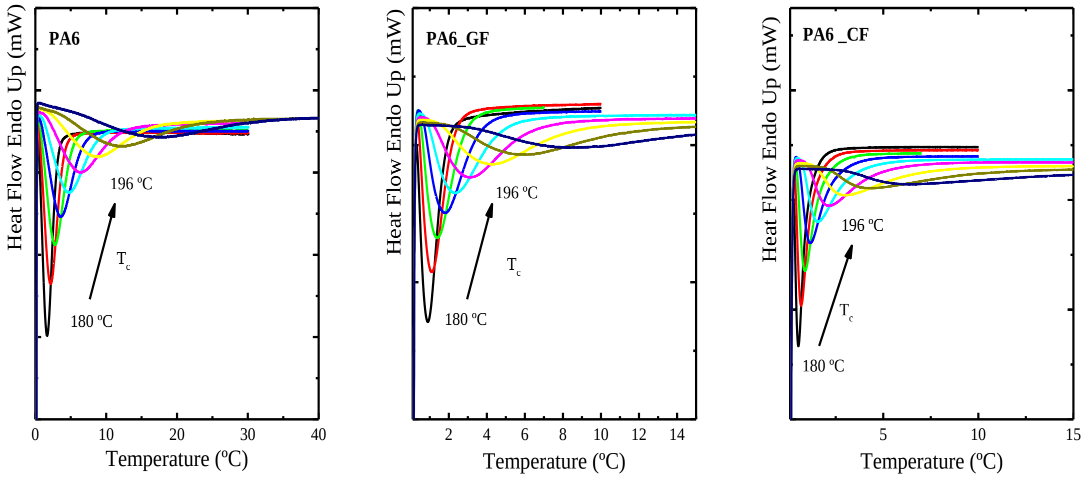

Figure SI 1. Isothermal crystallization exotherms of neat PA6 and for the glass and carbon fiber composites.

Table SI 1. Data obtained using Avrami equation.

**PA6**	***n***	**K (min^{-n})**	**$\tau_{50\%\ theo}$ (min)**	**$\tau_{50\%\ exp}$ (min)**	
**180 °C**	2.29	2.88E-1	1.468	1.415	0.9998
**182 °C**	2.22	1.71E-1	1.879	1.802	0.9998
**184 °C**	2.16	1.05E-1	2.391	2.29	0.9998
**186 °C**	2.15	6.42E-2	3.029	2.927	0.9999
**188 °C**	2.09	4.19E-2	3.816	3.662	0.9999
**190 °C**	2.11	2.20E-2	5.128	4.997	1
**192 °C**	2.1	1.21E-2	6.878	6.702	0.9999
**194 °C**	2.09	6.10E-3	9.597	9.37	0.9999
**196 °C**	2.04	3.27E-3	13.74	13.74	1

**PA6-GF**	***n***	**K (min^{-n})**	**$\tau_{50\%\ theo}$ (min)**	**$\tau_{50\%\ exp}$ (min)**	**R^2**
**180 °C**	2.23	1.74	0.661	0.673	0.9999
**182 °C**	2.22	9.18E-1	0.881	0.907	0.9999
**184 °C**	2.19	5.80E-1	1.085	1.108	0.9999
**186 °C**	2.27	2.92E-1	1.463	1.513	0.9999
**188 °C**	2.24	1.71E-1	1.869	1.924	0.9999
**190 °C**	2.26	8.70E-2	2.501	2.574	0.9999
**192 °C**	2.29	4.37E-2	3.349	3.432	1
**194 °C**	2.22	2.60E-2	4.402	4.477	1
**196 °C**	2.30	6.84E-3	6.589	6.726	1

**PA6-CF**	***n***	**K (min^{-n})**	**$\tau_{50\%\ theo}$ (min)**	**$\tau_{50\%\ exp}$ (min)**	**R^2**
**180 °C**	2.61	1.15E1	0.34	0.37	0.9997
**182 °C**	2.57	5.10	0.46	0.497	0.9998
**184 °C**	2.59	1.89	0.678	0.755	0.9996
**186 °C**	2.46	8.48E-1	0.921	1.021	0.9997

**188 ºC**	2.28	4.84E-1	1.171	1.277	0.9998
**190 ºC**	2.22	2.39E-1	1.617	1.767	0.9998
**192 ºC**	2.06	1.57E-1	2.057	2.206	0.9999
**194 ºC**	2.3	3.89E-2	3.493	3.917	0.9996
**196 ºC**	2.17	2.37E-2	4.856	5.377	0.9996
